# No Sex Differences in the Amount of Type IV Collagen in Wistar Rats Regardless of Sampling Strategy

**DOI:** 10.1111/apm.70236

**Published:** 2026-07-13

**Authors:** Patrik Mik, Katsiaryna Barannikava, Polina Surkova, Richard Pálek, Jáchym Rosendorf, Štěpánka Jansová, Václav Liška, Zbyněk Tonar

**Affiliations:** ^1^ Department of Anatomy Faculty of Medicine in Pilsen, Charles University Pilsen Czech Republic; ^2^ Department of Histology and Embryology Faculty of Medicine in Pilsen, Charles University Pilsen Czech Republic; ^3^ Biomedical Center, Faculty of Medicine in Pilsen, Charles University Pilsen Czech Republic; ^4^ Department of Surgery University Hospital in Pilsen Pilsen Czech Republic

**Keywords:** collagen type IV, fibrosis, liver diseases, stereology

## Abstract

Serum collagen IV (Col IV) is an important marker for staging liver fibrosis, but histological baselines in healthy tissue are often lacking or biased. This study applied unbiased stereological sampling to quantify liver Col IV in healthy adult Wistar rats and assess sex differences. No significant differences were observed between sampling strategies (simple, systematic, or stratified random) or between males (4.26% ± 0.93%) and females (3.71% ± 1.27%), yielding a pooled mean of 3.96% ± 1.11%. The Col IV‐positive area (3.62% ± 1.29%) did not differ significantly from the total collagen area measured by Sirius red (3.06% ± 1.72%), although a statistical trend was noted (R^2^ = 0.5845, *p* = 0.0767). These data indicate that IHC‐based area measurements capture a larger structural footprint of the mesh‐like Col IV network than biochemical mass‐based methods, and that 58% of the variance in Col IV area is explained by Sirius red‐positive area, suggesting a regulated spatial balance. The absence of sex effects on Col IV implies that previously reported sex‐related differences in total liver collagen likely involve other collagen types. Unbiased Col IV quantification may support validation of minimally invasive methods for staging liver fibrosis.

## 1 Introduction

Type IV collagen (Col IV) is a non‐fibrillar network‐forming protein made up of up to six genetically distinct α‐chains (α1 to α6) [1]. It is found in three isoforms, exclusively in the lamina densa of the basal lamina; the α1(IV)_2_α2(IV) isoform is almost the only form found in the healthy liver [2, 3]. In the Col IV α‐chain, two non‐collagenous domains can be described at the ends: A short N‐terminal 7S domain, a C‐terminal globular NC1 domain, and a long middle triple helical collagenous domain. For the respective components of Col IV, it has been suggested that serum levels of the 7S domain and NC1 domain probably reflect basement membrane degradation, whereas the levels of the Col IV helical region represent basement membrane formation [3]. Due to the activity of Col IV promoters, Col IV assembles into a superstructure that resembles a polygonal lattice, and in the ensemble of 21 other healthy liver basement membrane proteins, it can assemble to form a functional basement membrane [2]. In contrast to the normal functional basement membrane, which is present around large blood vessels in portal tracts, the basement layer found in the space of Disse is much less dense and contains fenestrations that facilitate the exchange of substances between the sinusoidal blood and hepatocytes [2]. This arrangement is probably due to the absence of laminin and nidogen in the extracellular matrix surrounding mature hepatocytes, and, along with E‐cadherin, adherens junctions, and lipid rafts, is among the key factors in establishing and maintaining hepatocyte membrane polarization [4]. Moreover, the unique liver Col IV isoform composition is important for maintaining hepatocyte viability [5].

However, with age and in various chronic liver diseases, the sinusoidal fenestrae can be lost, followed by basement membrane formation and marked by fibrogenesis in the space of Disse [6]. The process of losing the fenestrae has been termed the capillarization of hepatic sinusoids [7]. In the capillarized sinusoids, the bidirectional exchange of various substances is compromised, leading to hepatic dysfunction.

The fibrogenesis has been reported to differ in many chronic liver diseases, with a similar incidence between males and females [8]. In the case of Metabolically Dysfunctional‐Associated Fatty Liver Disease (MAFLD), sex is an important factor that favors males [9], for estrogen may play an important fibroprotective role in the progression of MAFLD [10]. Moreover, sexual dimorphism has been reported even in healthy livers in laboratory animals [11, 12]. There is some indication that the same is true for humans, as liver stiffness values were reported to be larger in healthy males [13, 14]. Therefore, the amount of liver connective tissue seems to be greater in males as a baseline condition.

Unfortunately, the quantification of the amount of liver connective tissue in biomedical research experiments has raised several issues. In our recent meta‐analysis of 54 studies [15] examining the variability in the amount of liver connective tissue in healthy rat liver, we found up to 170‐fold differences in the amount of total liver connective tissue among both Wistar and Sprague‐Dawley rats. Significant differences were found even within individual studies that included multiple rat groups. Moreover, in 86% of the studies, neither the original location of the tissue sample nor the tissue sampling process was specified to the extent for potential replication of the research. Only seven of the studies (14%) stated at least the original liver lobe of the sample. These issues disqualify the data generated from comparative studies or from evaluations of non‐invasive methods of liver fibrosis. In addition, incorrect handling of liver fibrosis scoring was also an issue. As a result, so far, only the amount of liver Col I and III (stained with Sirius red) has been quantified using unbiased and reproducible methodology. Consequently, our goal was to assess the variability in the amount of Col IV among different unbiased sampling strategies and to assess sexual dimorphism in the amount of rat liver Col IV.

## 2 Materials and Methods

### 2.1 Animals and Tissue Processing

For this purpose, healthy livers were harvested from 12 adult Wistar rats (six males, six females), aged six to seven months; the males weighing 433 ± 20 g, with a body length of 25.6 ± 0.5 cm, the females weighing 294 ± 19 g, with a body length of 23 ± 0.9 cm. Initially, the animals were anesthetized with an intramuscular injection of xylazine and ketamine. Once the depth of anesthesia was confirmed, a transversal laparotomy was performed. The infrahepatic part of the inferior vena cava was exposed, and a blood sample was collected. Ligaments supporting the liver were cut, followed by transection of the suprahepatic and infrahepatic inferior vena cava as well as the hepatoduodenal ligament. The liver was then removed, and the animal was immediately sacrificed by cervical dislocation. Following euthanasia, each liver was extracted and fixed in a 4% formaldehyde neutral buffered solution. One male rat exhibited situs inversus totalis with a diaphragmatic hernia, so this liver was not included in the analysis.

### 2.2 Sampling

**FIGURE 1:**
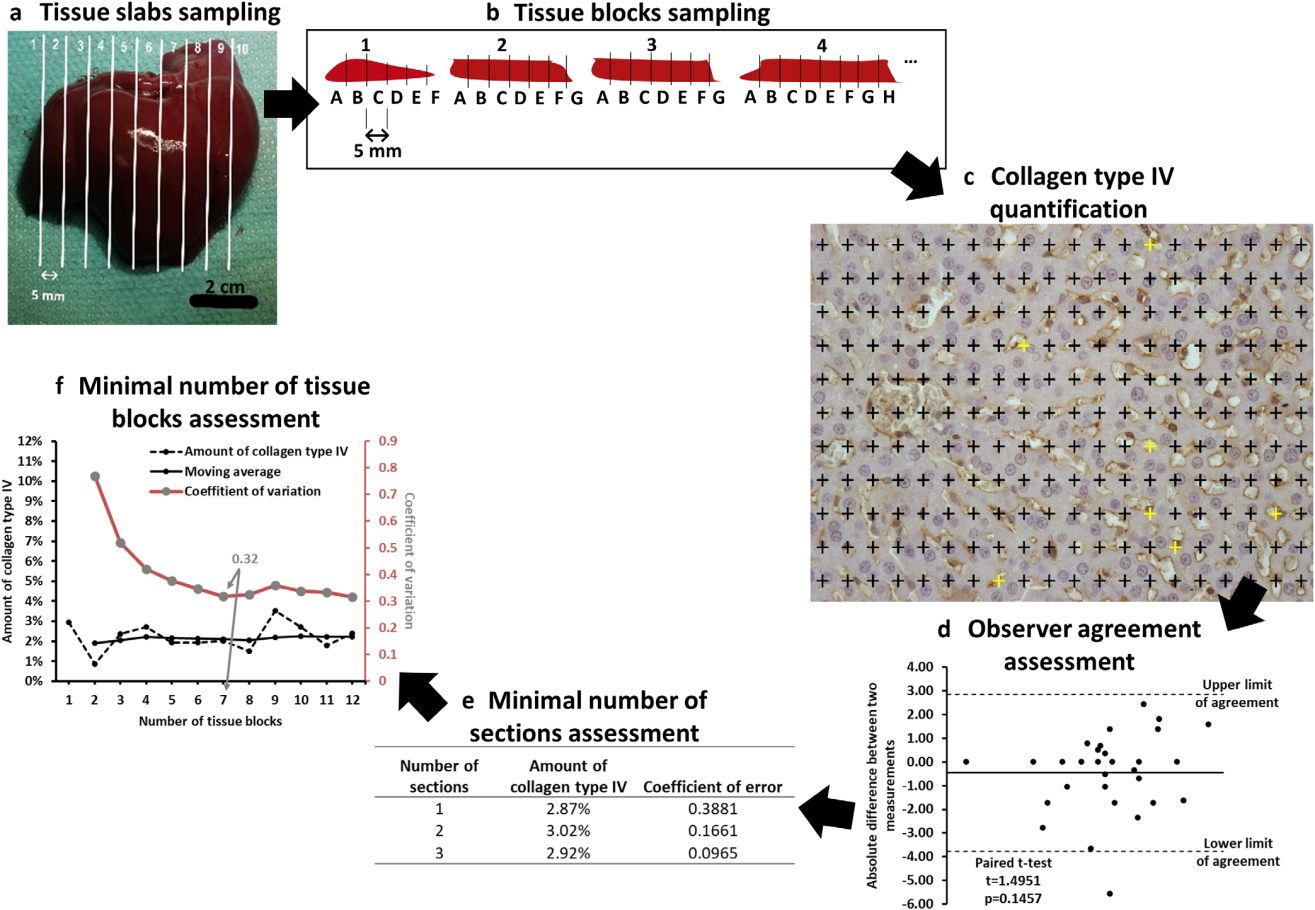
A flowchart of the sampling optimization and quantification process. In step (a), the liver is cut thoroughly into 0.5 cm‐thick slabs in the sagittal anatomical plane. Then, in step (b), each slab is cut perpendicularly (i.e., in the transverse plane) into 0.5 cm tissue blocks labeled A, B, C, etc. One tissue block from each slab is randomly selected for further quantification optimization. In step (c), the area fraction of Col IV is quantified using a point grid and point counting method. A point grid is overlaid on a microphotograph of the field of view (FOV), and the positive “hits”‐intersections of the point grid with Col IV positive structures (indicated by yellow crosses) are counted and divided by the total number of points on the grid. This provides the relative Col IV positive area. For intra‐observer agreement analysis in step (d), 20 FOVs were quantified twice by the same researcher after a one‐month interval, and the data were analyzed to assess the agreement. Next, in step (e), multiple histological sections from one tissue block are quantified to evaluate the relationship of the coefficient of error to the number of sections. It is determined that a minimum of three sections per tissue block is needed. In step (f), the minimal number of tissue blocks required is assessed by analyzing the moving average and coefficient of variability. The coefficient is smallest (32%) when seven blocks are used and does not decrease further. Additionally, the moving average does not change significantly beyond this point.

**FIGURE 2:**
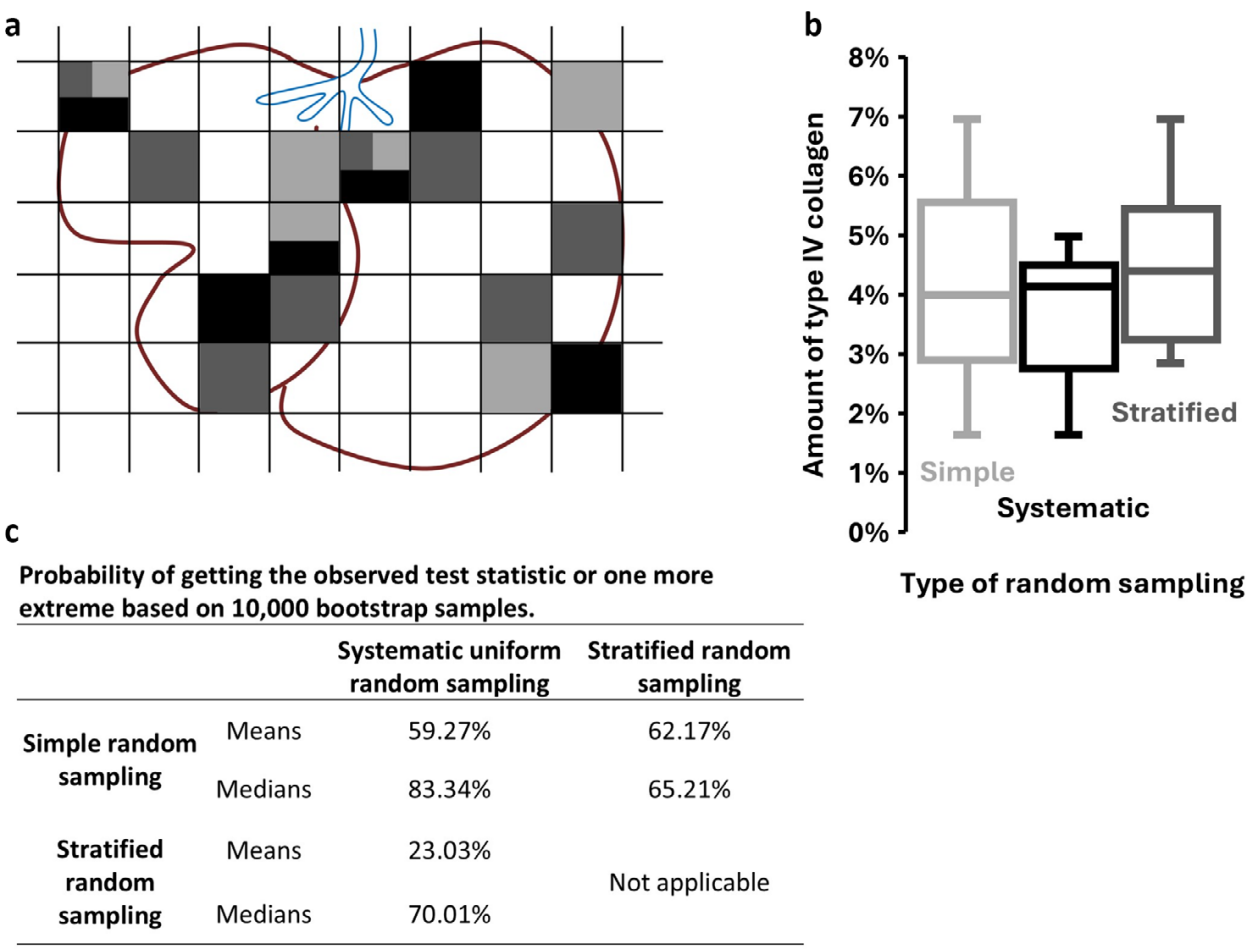
An assessment of the differences among three different sampling strategies. (a) An example of sampling using different strategies: After hepatectomy, each liver was cut into 0.5 cm‐thick slabs in the sagittal anatomical plane (vertical black lines). Each slab was then cut into 0.5 cm tissue blocks (horizontal black lines). Tissue blocks selected through simple random sampling are depicted in light gray, systematic uniform random tissue blocks in black, and tissue blocks sampled through stratified random sampling (our sampling) strategy in dark gray. Where colors overlap, the tissue block was used in corresponding sampling strategies based on the colors used. (b) Boxplots showing the amounts of liver collagen type IV when comparing the results of simple random sampling and systematic uniform random sampling. (c) Differences among the sampling strategies were assessed using a bootstrapping approach. We present the readers with the probabilities of obtaining the observed statistics or a more extreme result using 10,000 bootstrap resamples. This probability corresponds to a *p*‐value. As shown, none of the differences were statistically significant.

Each liver was thoroughly cut into 0.5 cm thick slabs in the sagittal anatomical plane (Figure 1a). Subsequently, each slab was cut perpendicularly (i.e., in the transverse plane) into 0.5 cm tissue blocks (Figure 1b). One tissue block from each slab was randomly selected for further quantification. Prior to cutting, the orientation of each tissue block was randomized using the orientator scheme to produce isotropic, uniform random histological sections for future use of the same blocks to test other hypotheses. Additionally, tissue blocks were sampled from one randomly selected rat using both simple random sampling and uniform random sampling to compare different sampling strategies (Figure 2a).

### 2.3 Immunohistochemical Staining

To differentiate Col IV, we followed standard histological procedures to process the tissue blocks into 4 μm‐thick paraffin‐embedded sections. After cutting, the specimens were deparaffinized in xylene and alcohol, and then hydrated with distilled water. Antigens were retrieved using proteinase K (5 min at 25°C). For the blocking procedure, we used a 30‐min peroxidase blocking method, washed twice for five minutes each with TBS + 0.025% Triton X‐100, and incubated for two hours in 10% Goat serum + 1% BSA (bovine albumin serum) in TBS at room temperature. The sections were then treated with primary antibodies (collagen type IV, Abcam, 1:1500) for 18 h at 4°C, rinsed twice for five minutes each with TBS containing 0.025% Triton X‐100, and incubated for 30 min with secondary antibodies (N‐Histofine Simple Stain MOX PO, Nichirei Biosciences Inc.). This was followed by three two‐minute rinses with Wash buffer, staining with DAB reagent, and a five‐minute wash under running water.

To ensure the specificity of the immunoreaction, negative controls were performed by omitting the primary antibody and replacing it with phosphate‐buffered saline (PBS) during the staining run. These sections demonstrated a total absence of specific staining (see Figure S1). Furthermore, the basement membranes of branches of the portal vessels present within the liver sections served as internal positive controls, showing consistent, strong immunoreactivity that verified the technical performance and analytical sensitivity of the procedure.

### 2.4 Quantification

For the quantification of the amount of Col IV, three different sampling strategies were used in the first step to assess differences based on the sampling scheme used. To quantify the area fraction of Col IV in each histological section, we initially sampled microscopic fields of view (FOVs) in a uniform random manner to represent approximately 10% of the section area. All FOVs were captured at 20× magnification with a resolution of 1280 × 960 pixels. In the second step, a point grid was applied over the FOVs using Ellipse software ViDiTo, Kosice, Slovak Republic [12]; to quantify the area fraction of Col IV using the point counting method (Figure 1c). The fibrous capsule of the liver, if present, was excluded from the quantification. Tissue processing artifacts, such as microcracks or folds, were not considered and were subtracted from the reference space. In one randomly selected rat, we compared the results of stratified random sampling (our sampling strategy) to other possible sampling strategies—systematic uniform random sampling and simple random sampling. The reliability of the quantification (repeated measurements) was assessed on 20 randomly selected fields of view (Figure 1d). To ensure a reliable quantification, optimization of the process was necessary. To validate our findings, we additionally stained successive sections from one rat liver with Sirius red and quantified connective tissue under polarized light. The reference area (total tissue) was quantified under the bright‐field illumination.

The optimization should be done in reverse to the process of tissue sampling and further quantification. Initially, the optimal number of histological sections must be determined, followed by an assessment of the optimal number of tissue blocks. The variability among histological sections was evaluated using the coefficient of error analysis (Figure 1e). The minimal number of tissue blocks was adjusted based on moving average analysis and changes in the coefficient of variability (Figure 1f). This led to a minimum of three sections per tissue block (coefficient of error = 0.0965) and seven tissue blocks (coefficient of variation 0.32) per liver sample. Therefore, the quantification using light microscopy was conducted on 1345 fields of view, using 294 histological sections (98 tissue blocks, three sections per block). The reliability of the quantification (repeated measurements by one observer) was tested on 20 randomly selected fields of view (Figure 1d).

### 2.5 Statistical Analysis

Repeated quantification (intra‐observer variability) was analyzed using a Bland–Altman graph and paired *t*‐test to assess the reliability of the quantification. To compare different sampling strategies, we employed a bootstrapping approach since the samples were not independent and did not have equal sizes. The bootstrap analysis was conducted using the software R (R Foundation for Statistical Computing, Vienna, Austria). Differences between the two sexes were analyzed using an unpaired *t*‐test, with the assumptions for the test being met.

### 2.6 Artificial Intelligence Generated Content

The English editing of this manuscript was assisted by a large language model (ChatGPT).

## 3 Results

**FIGURE 3:**
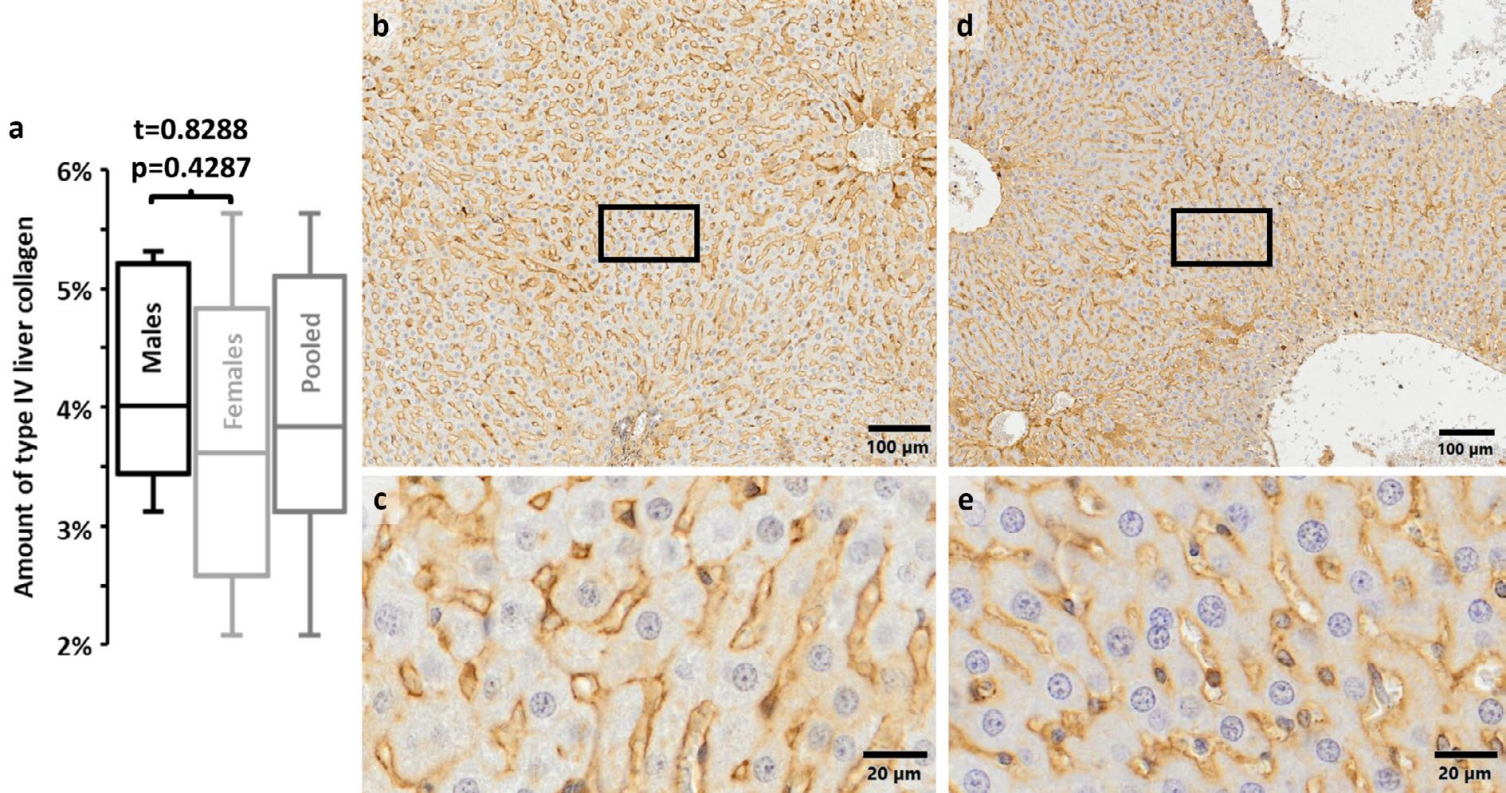
The results of the collagen type IV quantification. (a) Boxplots display the amount of collagen type IV, and further statistical analysis (*t*‐test) did not reveal any sexual differences in the amount of Col IV. (b, c) Representative images of normal rat male liver parenchyma, immunohistochemically stained for collagen type IV (brown color). The black rectangle in (b) indicates the area of the higher magnification image in (c). (d, e) Representative images of normal rat female liver parenchyma, immunohistochemically stained for collagen type IV (brown color). The black rectangle in (d) indicates the area of the higher magnification image in (e). As shown in (b–d), the brown staining was predominantly present along the sides of liver sinusoids, marking the lamina densa of the subtle basal lamina even in healthy rat liver parenchyma.

The Bland–Altman graph revealed only one significantly deviated difference between repeated quantification, with limits of agreement between 2.85% and −3.77%. The paired *t*‐test was not significant at α = 0.05 (t = 1.4951, *p* = 0.1457) (Figure 1d). No significant differences were found among the mean amounts of Col IV quantified when using different random sampling strategies—4.16% ± 1.80% and 3.99%, 3.38%–4.92% (mean ± SD and median, Q1–Q3) in the simple random sampling, 3.73% ± 1.19% and 4.14%, 3.34%–4.33% in the systematic uniform random sampling, and 4.57% ± 1.40% and 4.40%, 3.62%–5.27% in stratified random sampling (our sampling) (Figure 2b). We also did not observe significant differences in the liver Col IV between healthy albino male and female rats (Figure 3a). The pooled mean amount of Col IV was 3.96% ± 1.11% (mean ± SD), 4.26% ± 0.93% in males, and 3.71% ± 1.27% in females (Figure 3a). The observed differences were not significant (t = 0.8288, *p* = 0.4287) (Figure 3a). On the other hand, a large variability in the amount of Col IV was observed; the coefficient of variation was over 20% (28% in the pooled sample; 21.83% in the male rats; 34.23% in the female rats). Descriptive statistics are summarized in Table 1. Representative images of the observed amounts are depicted in Figure 3 for both males (Figure 3b,c) and females (Figure 3d,e). The mean Sirius red–positive area (3.06% ± 1.72%) did not differ from the Col IV–positive area (3.62% ± 1.29%), although a negative association was observed between them (R^2^ = 0.5845, *p* = 0.0767).

**TABLE 1:** Descriptive statistics of the amount of rat liver collagen type IV.

Sex	Amount of rat liver collagen IV				
	Q1	Median	Q3	Mean	SD
Males	3.76%	4.01%	5.11%	4.26%	0.93%
Females	2.91%	3.62%	4.38%	3.71%	1.27%
Pooled	3.26%	3.84%	4.84%	3.96%	1.11%

## 4 Discussion

We did not find significant differences in the amount of hepatic Col IV between healthy male and female albino rats. This is in contrast with previously published results of the quantification of liver connective tissue. Marcos and colleagues [11, 16] found significant sex differences in healthy adult and old rats, with females having significantly less connective tissue. Our previous study on pig livers also showed that females have significantly less connective tissue when compared to males [12]. No other unbiased results on sex differences in the amount of liver connective tissue have been published so far. Since we did not analyze levels of sex hormones in this study, their role in the Col IV deposition or degradation cannot be directly addressed. Moreover, Col IV represents only a minor fraction of hepatic collagen compared with fibrillar Col I and III. It is therefore plausible that previously reported sex‐related differences in total liver collagen primarily reflect changes in other collagen types rather than in Col IV.

As previously demonstrated, there have been reports of up to a 170‐fold difference in the amount of liver connective tissue in healthy rats. Unfortunately, most studies did not adhere to unbiased methodology [15] leaving the expected variability of liver connective tissue among rats unknown. The only unbiased quantifications of the amount of healthy rat liver connective tissue we found ranged from 0.50% to 3.2% [11, 16, 17, 18]. Interestingly, Marcos and Correia‐Gomes [19] observed the amount of liver connective tissue in healthy male rats to vary significantly between 2.0% and 3.2% based on age, likely due to an increase in liver collagen along liver sinusoids (Sirius red staining). This was probably due to a relatively larger increase in the amount of liver collagen along liver sinusoids when compared to other parts of the liver lobule [19], potentially linked to capillarization of liver sinusoids as seen in elderly livers [20]. Therefore, the age of laboratory animals has to be controlled for when planning an experiment involving the histological quantification of liver fibrosis.

In addition to age, the potential influence of biological sex on liver architecture must be considered. In this regard, our findings of uniform Col IV amounts across sexes are consistent with the stereological work of Marcos and Correia‐Gomes [19], who reported that the porto‐central distance remains stable between male and female Wistar rats at 6 months of age. This finding indicates that during early adulthood, the hepatic scaffold exhibits a high degree of architectural symmetry between sexes, both in terms of its macro‐structural dimensions and its basement membrane composition. This observation, despite the significant coefficient of variation observed in the samples, lends further credence to the hypothesis that, while individual biological heterogeneity is indeed substantial, the overall structural “footprint” of the liver lobule remains balanced. The absence of any statistically significant differences in both Col IV and porto‐central distance between the two groups suggests that pathological changes in these metrics can be interpreted as genuine departures from homeostasis rather than physiological variations. As the rats age towards 18 months, many of the cytological differences, such as the number per gram of hepatocytes and Kupffer cells, begin to disappear. However, the porto‐central distance remains a stable anchor throughout this transition, thereby ensuring the physical geometry of the lobule is preserved [19]. While the macro‐structure is identical, as previously demonstrated in the porcine liver, the cellular populations are heterogeneous: Males have larger mononuclear hepatocytes, while females have higher hepatocellularity and more binucleated hepatocytes [21]. These data provide further indication that the peripheral (subcapsular) regions of hepatic lobes contain the largest mononuclear hepatocytes with the smallest numerical density, while cells in proximity to the paracaval or paraportal regions are smaller. This finding suggests that environmental factors or proximity to major vasculature may influence cellular growth and ploidy. These varying cellular units are then arranged into a standardized porto‐central cord of 16 to 17 cells [19]. This finding suggests that the macro‐structural geometry of the liver is established early in development and is maintained consistently throughout life. In addition, the cellular composition of the liver varies in terms of size and nuclearity to meet the specific metabolic demands of each gender, whilst remaining within a shared structural framework.

Another key point concerns the unexpectedly high Col IV values in our study (mean 3.96% ± 1.11%), which appear above the commonly reported range for total liver connective tissue. We acknowledge that previous studies e.g., [22], quantified Col I, III, and “other collagens,” without specific data on Col IV. The only earlier quantitative report we found was Aycock and Seyer [23], later complemented by Arteel and Naba [2], which together suggest Col IV represents about one‐seventh the abundance of Col I. Depending on the methodology, Col IV estimates ranged from 6.9% to 7.7% of total collagen, consistent with a six‐ to sevenfold lower abundance than Col I, and about 10‐fold lower when compared with the combined amount of Col I and III.

Our findings, however, demonstrate that the Col IV‐positive area (3.62% ± 1.29%) is statistically comparable to the total collagen area measured by Sirius Red (3.06% ± 1.72%). The fact that Col IV occupies an area similar to the total collagen pool suggests that its spatial distribution in the liver is much more extensive than its relative mass would imply. These discrepancies may arise from methodological differences. Biochemical and proteomic studies quantify collagen mass, which tends to underrepresent network‐forming collagens such as type IV. In contrast, immunohistochemistry (IHC) measures the area occupied by collagen molecules. Because Col IV forms a mesh‐like network, it occupies more space per unit mass than fibrillar Col I and III. Thus, IHC‐based estimates of Col IV are expected to be higher than mass‐based quantifications. Moreover, studies using X‐ray diffraction and biochemical quantification have shown that the packing density and volume fraction of collagen fibrils can vary between types and tissues. For example, in the corneal stroma, the intermolecular Bragg spacing is highly conserved, but the overall fibril diameter and interfibrillar spacing vary, leading to differences in volume fraction [24]. In articular cartilage, the relative abundance and organization of Col II, IX, and XI differ from those in tendon or bone, which are rich in Col I [25]. Further, the effective volume occupied by collagen is also influenced by tissue hydration, crosslinking, and the presence of other ECM components such as glycosaminoglycans and proteoglycans [24, 26]. Since unbiased volumetric data for Col IV in healthy rat liver are lacking, relatively high estimates cannot be excluded.

Furthermore, the relationship between the Sirius Red and Col IV‐positive area was found to be negatively associated (R^2^ = 0.5845, *p* = 0.0767). In the context of conventional null‐hypothesis testing, a *p*‐value of 0.0767 is frequently disregarded as “not significant”. However, the R2 value of 0.5845 indicates that approximately 58.5% of the variance in the Col IV‐positive area can be explained by the variance in the Sirius Red‐positive area. This moderate‐to‐strong correlation serves to reinforce the concept that these two collagen compartments do not fluctuate independently but rather constitute part of a regulated structural system. To provide a hypothetical example, in the event of a localized increase in fibrillar collagen—which could be considered a minor adaptive response to hemodynamic fluctuations—the resulting increase in mechanical tension or ligand density may trigger a metabolic downregulation of network‐forming components such as Col IV.

### 4.1 Limitations

Although we followed a rigorous methodology, we acknowledge some limitations of our study. First, we did not account for the tissue shrinkage. In the pig liver, it has been found that shrinkage is not isotropic in the liver parenchyma [21]. Second, while we did not observe any histopathological alterations, we did not conduct liver biochemical tests or liver function tests in this study. Third, the levels of sex hormones were not analyzed; therefore, the interpretation of our results regarding the sex of the animals is limited. Fourth, while our negative controls (primary antibody omission) confirmed the absence of non‐specific binding from the detection system, we did not perform a secondary negative control using an irrelevant protein solution, such as bovine serum albumin (BSA), in place of the primary antibody. However, the high signal‐to‐noise ratio observed—characterized by sharp sinusoid labeling against a clear parenchyma in the negative controls—suggests minimal impact on the final quantification.

### 4.2 Future Application of Our Findings

The relative abundance of collagen types in total liver collagen was initially reported to be 42.5% (type I), 39.5% (type III), 10.6% (type V), 6.9% (type IV), and 0.6% (type VI) [23]. Recently, mass spectrometry in healthy human liver revealed that the relative abundance of Col IV is approximately seven times lower than Col I, more than two times lower than Col III, two times lower than Col VI, and two times higher than Col V (thus, the abundance of collagen types I > III>VI > IV > V) [2] In the context of liver pathology, the ratio of Col IV to other collagen types also becomes important. In liver fibrosis, the collagen content progressively increases following a non‐linear trend [27]. This non‐linear increase has also been confirmed specifically for Col IV in MAFLD [28] or in alcohol‐associated liver disease [29]. In liver disease, the relative abundance of liver collagen types changes. For example, in alcoholic liver cirrhosis, it was reported to be 56.6% (type I), 28.0% (type III), 9.6% (type V), 5.5% (type IV), and 0.5% (type VI) (I > III > V > IV> VI). Importantly, changes in the amount of Col IV have been observed in hepatic injury of different etiologies. Therefore, Col IV or its 7S domain has been suggested as a reliable marker of fibrosis staging in MAFLD [30], hepatitis B virus infection [31], and hepatitis C virus infection [32] Col IV levels are also associated with hepatocellular injury in cholestasis [33]. The applicability of serum Col IV for staging in alcohol‐associated liver disease and primary biliary cirrhosis remains to be resolved. Therefore, the unbiased quantification of liver Col IV could provide us with unbiased data for validation of minimally invasive methods for staging early liver fibrosis. For planning an experiment involving the amount of liver Col IV, we provide the minimum detectable effect according to Vittinghoff et al. [34] and the minimum sample size needed for the detection of such an effect [35] in Table 2. However, future research involving the histological quantification of liver fibrosis should follow an unbiased sampling strategy e.g., [15, 36], and control for the age of experimental animals.

**TABLE 2:** The minimum detectable effect according to Vittinghoff et al. [29]. This information indicates the smallest measurable increase in the amount of Col IV that our data can detect. Additionally, the analysis according to Ahn et al. [30]. reveals the sample size required to detect such an increase.

Sex	Minimum detectable effect [29, 34]	Minimum number of animals needed for this detection [30, 35]
Males	1.16% (4.26% → 5.42%)	5
Females	1.46% (3.71% → 5.17%)	6
Pooled	1.04% (3.96% → 5.00%)	9

Although we focused on random stereological sampling, we recognize that some studies use lobe‐specific strategies, such as reserving one lobe for histology and others for different analyses. Because rat liver lobes overlap in two anatomical levels, dissecting them prior to sampling would have introduced a risk of bias. Our approach, which exhaustively cut the liver without regard to lobar anatomy, occasionally resulted in tissue blocks containing more than one lobe. As an indirect check, we compared tissue blocks from the extremes of the liver and found no meaningful differences. In line with this, our earlier work in pigs demonstrated that the position of tissue relative to vascular architecture exerted a stronger influence on collagen distribution than lobar identity, though inter‐lobar differences were still present [12]. Future studies might therefore combine lobe‐specific sampling with stereological principles to further explore this issue.

## 5 Conclusion

In conclusion, we did not find significant differences in the amount of liver Col IV between male and female albino rats. While Col IV constitutes a minor fraction of total liver collagen, its spatial distribution differs from fibrillar collagens, which may explain discrepancies with previous biochemical studies. Therefore, other collagen types are probably responsible for the previously reported sexual differences in the amount of liver connective tissue. This interpretation is reinforced by the value of R^2^ = 0.5845, which suggests that nearly 59% of the variance in Col IV area is explained by the variance in Sirius red‐positive area, a substantial correlation that points to a regulated spatial balance between the basement membrane and interstitial components.

When quantifying liver Col IV or any other collagen type, as well as total collagen, it is essential to utilize one of the proposed unbiased sampling strategies. These findings establish a reliable baseline for future validation of minimally invasive methods of staging liver fibrosis and highlight the importance of considering methodological differences when comparing volumetric vs. mass‐based collagen measurements.

## Funding

This work was supported by Ministerstvo Zdravotnictví Ceské Republiky (AZV NU22J‐06‐00058).

## Ethics Statement

The organ harvesting protocol was approved and monitored by the Ministry of Education, Youth, and Sports of the Czech Republic (project No. MSMT‐27374/2011–30). All procedures involving animals were conducted in accordance with Czech Republic law, which aligns with European Union legislation.

## Conflicts of Interest

The authors declare no conflicts of interest.

## Supporting information


**Figure S1:** Representative negative control staining of adult Wistar rat liver sections. (A, B) To verify the specificity of the immunohistochemical reaction, negative controls were performed by omitting the primary antibody and replacing it with phosphate‐buffered saline (PBS) during the staining run. These representative images show a total absence of brown (DAB) chromogen within the liver parenchyma, hepatocytes, and vascular structures. Only the blue nuclear counterstain (hematoxylin) is visible. These results confirm that the secondary antibody and detection reagents did not produce non‐specific background or react with endogenous peroxidase activity, ensuring that the quantified area fractions in the study represent specific Collagen IV immunoreactivity. Scale bars = 100 μm.

## Data Availability

The data that support the findings of this study are available from the corresponding author upon reasonable request.
