# Supplementary material for: No Sex Differences in the Amount of Type IV Collagen in Wistar Rats Regardless of Sampling Strategy
Source: APMIS. 2026 Jul 13;134(7):e70236. doi: 10.1111/apm.70236 (PMC13358885; doi:10.1111/apm.70236)
Supplement: Supplementary file 1 — Figure S1: Representative negative control staining of adult Wistar rat liver sections. (A, B) To verify the specificity of the immunohistochemical reaction, negative controls were performed by omitting the primary antibody and replacing it with phosphate‐buffered saline (PBS) during the staining run. These representative images show a total absence of brown (DAB) chromogen within the liver parenchyma, hepatocytes, and vascular structures. Only the blue nuclear counterstain (hematoxylin) is visible. These results confirm that the secondary antibody and detection reagents did not produce non‐specific background or react with endogenous peroxidase activity, ensuring that the quantified area fractions in the study represent specific Collagen IV immunoreactivity. Scale bars = 100 μm. [file APM-134-0-s001.docx]

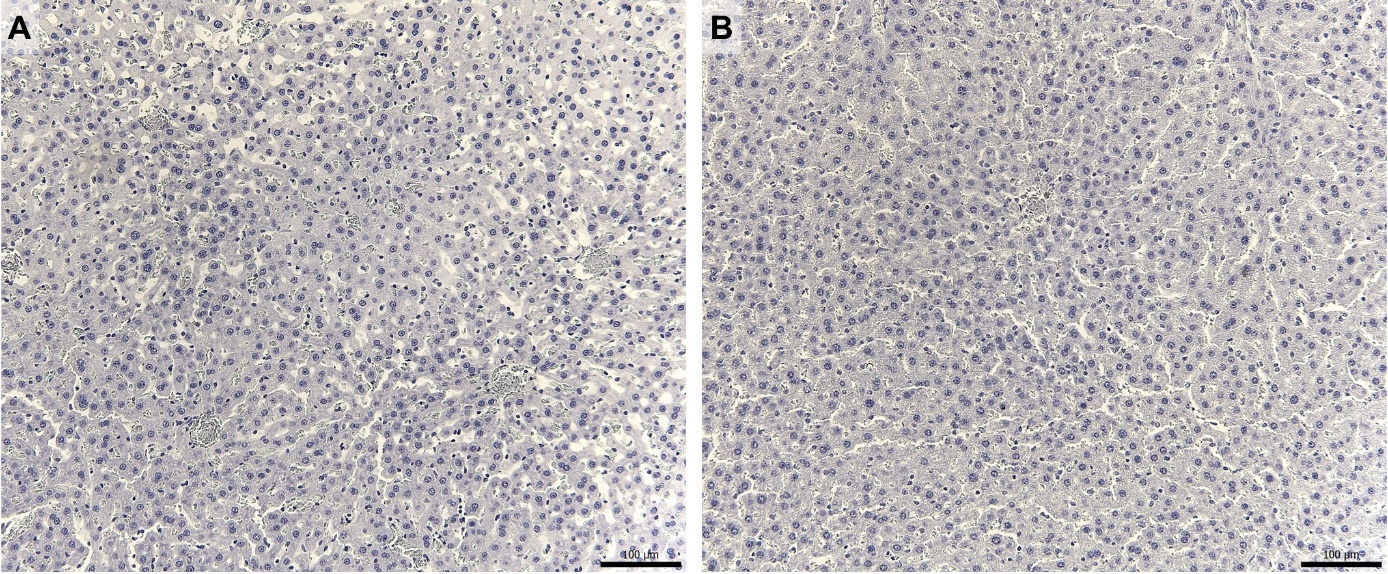


**Supplementary Figure 1.** **Representative negative control staining of adult Wistar rat liver sections. (A, B)** To verify the specificity of the immunohistochemical reaction, negative controls were performed by omitting the primary antibody and replacing it with phosphate-buffered saline (PBS) during the staining run. These representative images show a total absence of brown (DAB) chromogen within the liver parenchyma, hepatocytes, and vascular structures. Only the blue nuclear counterstain (hematoxylin) is visible. These results confirm that the secondary antibody and detection reagents did not produce non-specific background or react with endogenous peroxidase activity, ensuring that the quantified area fractions in the study represent specific Collagen IV immunoreactivity. Scale bars = 100 µm.
